# Multiple micronutrient supplementation improves growth and reduces the risk of anemia among infants in Gaza Strip, Palestine: a prospective randomized community trial

**DOI:** 10.1186/s12937-020-00652-7

**Published:** 2020-12-05

**Authors:** Ali Albelbeisi, Zalilah Mohd Shariff, Chan Yoke Mun, Hejar Abdul Rahman, Yehia Abed

**Affiliations:** 1 Department of Nutrition and Dietetics, Faculty of Medicine and Health Sciences, University Putra Malaysia, Seri Kembangan, Malaysia; 2 Department of Community Health, Faculty of Medicine and Health Sciences, University Putra Malaysia, Seri Kembangan, Malaysia; 3 Faculty of Public Health, Al Quds University of Gaza- Palestine, Jerusalem, Palestinian Territory

**Keywords:** Micronutrient supplementation, Child nutritional status, Physical growth, Hemoglobin, Palestine

## Abstract

**Background:**

Infants and young children 6–24 months of age are the most nutritionally vulnerable groups, as their needs for vitamins and minerals are high relative to the amount of food they consume. This study determines the effect of Micronutrient Powder Supplementation (MNP) on growth and hemoglobin of infants in Gaza Strip, Palestine.

**Method:**

This was a two-arm parallel-group randomized controlled trial conducted in two health care clinics of the United Nations Relief and Work Agency (UNRWA) at the Middle Area governorate of Gaza Strip, Palestine. A total of 200 healthy infants aged 6-month-old were recruited and randomized to receive 3 sachets/week of MNP for 12 months alongside with the National Micronutrient Supplement (NMS) (*n* = 100) or NMS alone (*n* = 100). Weight, length, blood hemoglobin, and dietary intakes were measured at 6, 9, 12, 15, 18, and 21 (3 months after the end of intervention) months of age. Analysis was by intention to treat.

**Results:**

The experimental group had a higher concentration of hemoglobin at 12 and 15 months than did the control group, and a significant difference (*p* <  0.05) was observed at 15 months only. Significantly greater weight, length, weight-for-age, length-for-age, and weight-for-length z-scores were observed in the experimental group than that in the control group at 12 and 15 months (*p* <  0.05). The prevalence of anemia (18% vs. 52%) and stunting (1% vs. 11%) were higher in the control than the experimental group, respectively. After controlling for sex, similar findings were reported.

**Conclusions:**

Addition of MNP to the existing NMS program improved the nutritional status of young children. The potential benefits of MNP supplementation on physical growth and hemoglobin should be considered in the existing NMS program.

**Trial registration:**

ISRCTN57594793; Date of registration: 20-03-2018 (Retrospectively registered).

## Introduction

Micronutrient deficiencies are reported as a global public health problem affecting 2 billion people in the developed and developing countries [1]. Deficiency of vitamins and minerals acts as exacerbating factors in chronic diseases, greatly impacting morbidity, mortality, and quality of life [2]. There is no global estimate of micronutrient deficiencies for under-two children, however, it has been reported that 190 and 293 million preschool children have vitamin A deficiency and anemia, respectively [3, 4]. In 2013, the Palestinian Micronutrient Survey (PMS) reported that vitamin A status was sufficient among 27% of under-five children only, and severe deficiency was more prevalent (36.8%) among Gaza Strip children [5] than those in the West bank. Moreover, the overall occurrence of anemia amongst preschool children in Gaza Strip was 59.7%, and the prevalence of mild and moderate anemia was 46.5, and 13.5%, respectively [6].

The main contributors to micronutrient deficiencies are lack of dietary diversity, poor bioavailability of minerals in plant foods, high incidence of infectious diseases, and increased physiological demands [7, 8]. Furthermore, deficiencies of vitamin A, iron, zinc, and iodine are common consequences of the cereal- and plant-based complementary foods typically introduced to infants and children in developing countries [9]. These foods are often low in energy and poor sources of bioavailable micronutrients particularly zinc and iron due to presence of phytate [8].

Micronutrient supplementation and food fortification have been shown to be the most cost-effective approaches to reduce micronutrient deficiencies among children [10]. Although the benefits of supplementation with single or multiple micronutrients are well-recognized, implementation has been hindered by poor compliance and adherence to the dosage regimens, inadequate coverage and supply, and potential side effects and safety concerns [11, 12]. In response to these constraints, at home food fortification with micronutrient powders (MNP) was promoted as a novel method to deliver micronutrients with foods. Several studies [13–15] found that MNP is effective in treating anemia and improving biochemical profile of micronutrients among infants and enhanced tolerability. Promising results were also found in recent studies using MNP with different combinations of micronutrients [16–18]. The use of MNP has obtained publicity due to ease of use and low cost [19] and it contains a lipid-encapsulated coating which prevents iron from dissolving directly into food and hence it does not change the color or taste of food or produce iron-related side effects [20]. MNP is a single dose packet of powder containing vital micronutrients that can be mixed onto any semi-solid food at home to enrich the food with the essential vitamins and minerals [21].

Although the Palestinian Ministry of Health (MOH) has implemented the National Micronutrient Supplementation program (NMS) since 2001 to improve the nutritional status of under-two children [22], anemia and growth retardation remain public health concerns in Gaza Strip. Radi et al., (2009) reported that 72.8, 34.3, 31.4, and 31.45% of children under two in Gaza city were anemic, wasted, stunted, and underweight, respectively [23]. This study was conducted to determine the effect of micronutrient supplement in powder form on physical growth and hemoglobin concentration of infants aged 6 months.

## Methods

### Study design and participants

This randomized controlled trial was conducted in two health care clinics of the UNRWA at the middle area governorate of Gaza Strip, Palestine. The Middle Area governorate has the highest percentage (86.5%) of refugees in the Gaza Strip, with a low socioeconomic status [24, 25]. The study was approved by the Helsinki Ethics Committee of Gaza Strip, as well as the Ethics Committee for Research Involving Human Subjects (JKEUPM) of the Faculty of Medicine and Health Sciences, Universiti Putra Malaysia. Permission to conduct the study in the UNRWA clinics was obtained from the Health Affairs Center of the UNRWA in Gaza Strip. This trial was carried out from October 2015 to January 2017.

**Fig. 1:**
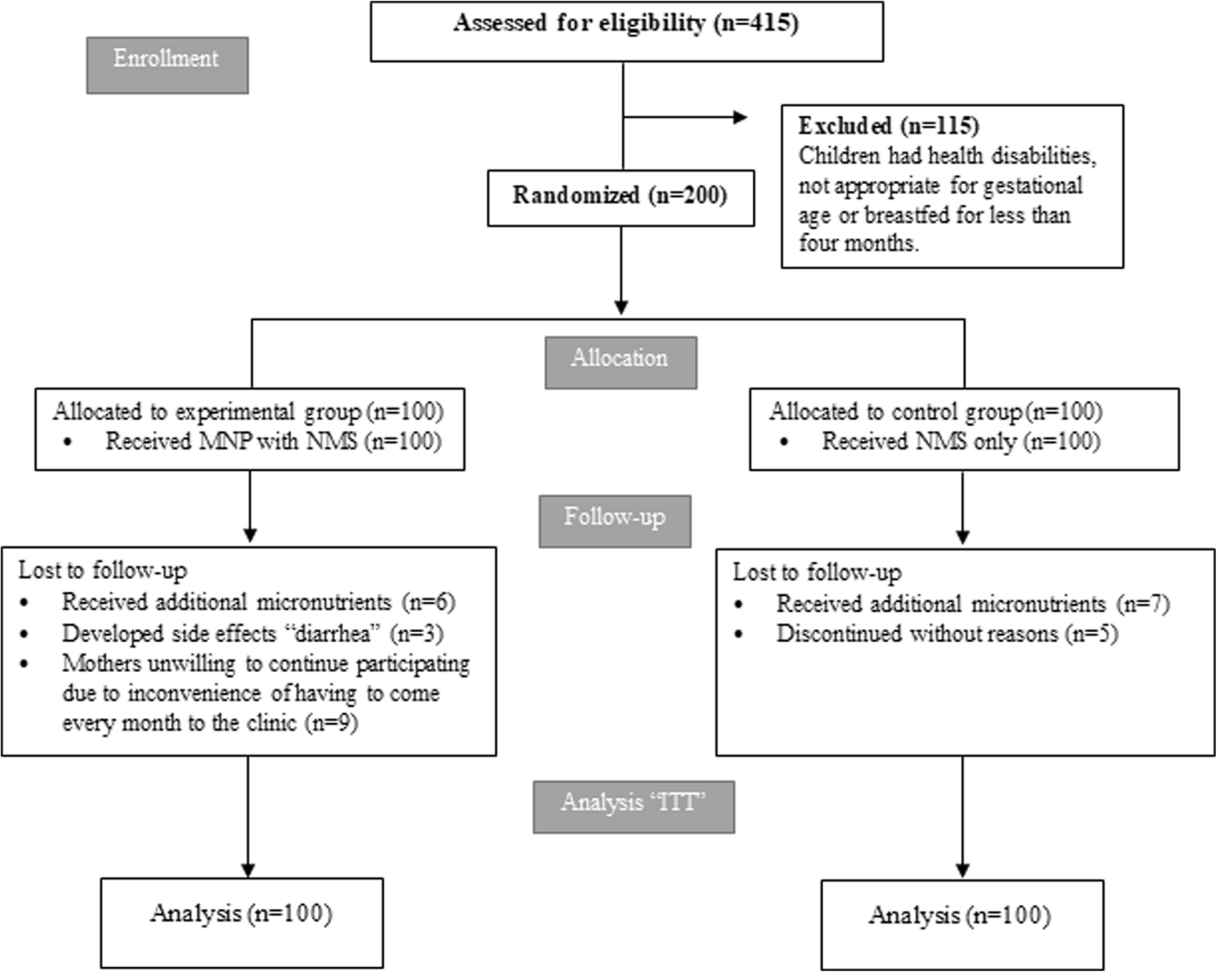
Flow diagram of subjects’ progress through the study

Sample size was calculated to detect a 0.7 cm difference in mean length between the experimental and control groups. Allowing a type I error level of 5% with power of 90% and assuming a dropout rate of 30%, 100 infants were required for each group. The total number of infants aged 5 months ± 2 weeks at the selected two clinics were 415. All infants were screened according to the study selection criteria ((healthy e.g. no physical disabilities or diseases, normal z scores (> -2 to < + 2 SD) of weight-for-age (WAZ), length-for-age (LAZ), and head circumference-for-age (HCZ), normal birth weight (≥ 2.5 to ≤4 kg), appropriate for gestational age (AGA), non-anemic, and breastfed for more than 4 months). Upon screening, a total of 300 infants were eligible for inclusion, and then infants’ parents were invited to give consent for their infants’ participation. They were informed about the purpose of the study, and only infants of parents who agreed with informed consent were included. Accordingly, 200 out of 300 eligible infants (108 males, 92 females) were randomly selected and allocated equally into two groups (Fig. 1).

Infants were randomly assigned to receive either the MNP with NMS or NMS only. MNP (Mix me™) was produced by DSM Nutritional Products Europe, Switzerland. Random allocation was performed with sealed opaque envelopes. Infant’s mother was asked to draw an envelope containing the allocation group number (1 for the experimental group and 2 for the control group). The study protocol scheduled administration of MNP for 12 months as 3 sachets per week (every other day). MNP were distributed to mothers monthly by two trained research assistants and given as one-dose sachets. MNP was packaged in a paper/aluminum/ polyethylene pouch with lot number and internal batch number printed on the package. Administration of MNP started immediately after baseline assessment. The NMS (Vitamin A, iron, and vitamin D) was provided by the UNRWA as part of the National Nutrition Supplementation program. Micronutrient composition and dosage of MNP and NMS are shown in Tables 1 and 2, respectively. The dose of vitamins and minerals was in accordance to the recommendation by UNICEF [29].

**Table 1:** Composition of the study supplement (MNP)

Micronutrient	Composition
Vitamin A	*400 μg*
Vitamin C	*30 mg*
Vitamin D	*5 μg*
Vitamin E	*5 mg α-TE*
Vitamin B1	*0.5 mg*
Vitamin B2	*0.5 mg*
Vitamin B6	*0.5 mg*
Vitamin B12	*0.9 μg*
Folic Acid	*90 μg*
Niacin	*6 mg*
Iron (Fumarate)	*10 mg*
Zinc	*4.1 mg*
Copper	*0.56 mg*
Selenium	17 μg
Iodine	*90* μg

Source: [26]

*DRI* Dietary reference intakes

**Table 2:** Composition and dosage of the NMS

Micronutrient	Composition	Onset (old)	Duration
Vitamin A	300–600 μg	9 months	One squeezed capsule repeated every 6 months.
Vitamin D	10 μg	9 months	2 drops for 30 days and repeated every 3 months.
Iron (Sulphate)	2 mg/kg/day	6 months	2 drops for12 months.

Source: [26–28]

*DRI* Dietary reference intakes

### Measurements

#### Primary and secondary outcomes

The primary outcomes were blood hemoglobin and growth parameters such as weight-for-age, length-for-age, and weight-for-length. Anemia was defined as mild (10 g/dl ≤ Hb < 11 g/dl), moderate (7 g/dl ≤ Hb < 10 g/dl), and severe (Hb < 7 g/dl). Underweight was defined as a weight-for-age z score < − 2·0; stunting was defined as a length-for-age z score < − 2·0; and wasting was defined as a weight-for-length z score < − 2·0. Energy and micronutrients intakes (iron, vitamin A, and vitamin D) as well as feeding practices were assessed as secondary outcomes and adequate intake of each dietary element was defined as greater or equal to 100% Estimated Average Requirements (EAR). Other measures such as demographic and socioeconomic data were also assessed.

#### Follow up and outcomes assessment

Two trained licensed nurses assessed infants at the clinics at baseline, 3-month intervals through the supplementation period (12 months), and 3 months after the end of supplementation. At baseline, information on socio-demographic, nutritional status, and health status of infants and their parents was gathered. At each visit, mothers were also interviewed about the general health status of infants and occurrence of adverse events such as vomiting, diarrhea, constipation, and discomfort. Based on a feasibility study undertaken by the author before the current study, compliance and acceptance of MNP as well as preference for at-home foods mixed with MNP were shown to be adequate [30].

The two nurses performed anthropometric assessments (every 3 months) using standard procedures [31] including body weight and length. Anthropometric measurements were taken in duplicate and were then averaged for each participant. The WHO ANTHRO program was used to calculate the z scores, and then the results were compared to the 2006 WHO growth reference [32]. Capillary blood (finger or heel prick) hemoglobin was measured at baseline, end of supplementation, and 3 months after the end of supplementation by the laboratory technician of the UNRWA clinic following the standard procedures established by the WHO [33] using spectrophotometer. Intakes of energy and micronutrients were assessed using a 1 day of 24-h diet recall at baseline, 6 months of supplementation, and at the end of supplementation period and the findings were compared to the EARs of under-three children to assess adequacy [26]. Nutritional data were analyzed by Nutritionist Pro™ software version 7.1.0 (Axxya Systems, USA).

### Statistical analysis

For all statistical tests, analysis was based on intention to treat; that is, the study results were analyzed using all available data on participants regardless of any protocol violation, such as imperfect adherence or contamination of treatment. Skewness and kurtosis values confirmed that data was normally distributed. The baseline comparison between the groups was done using the student’s t test. The Chi-square test was used to examine the association between two categorical variables. Repeated-measures analysis of variance (RMANOVA) was used to test for changes in growth, hemoglobin concentration, and energy and micronutrient intakes over time within and between groups, followed by Bonferroni correction for multiple comparisons. Subsequently, data were analyzed using analysis of covariance (ANCOVA) to adjust for sex. The results were considered significant when *p* ≤ 0.05. Practical clinical significant differences were determined by the effect size measured by the Standardized Mean Difference (SMD) (small = 0.2; medium = 0.5; large = 0.8). The SPSS software, version 23 (SPSS Inc., Chicago, IL) was used for the statistical analysis.

## Results

A total of 170 infants completed the supplementations (82 experimental group, 88 control group). The participation rate at the end of the study was 85% and mean overall compliance was 91.17% of the total intended MNP dose (empty/used MNP sachets were counted on monthly basis). There were no significant differences in baseline measurements between the children who completed the study and those who did not, as well as, no significant differences in feeding practices between the two groups were reported throughout the study period (Table 3). No adverse events were observed among participants throughout the study. As shown in Table 4, the baseline characteristics of the participants showed no significant difference between the two groups and all of participants had normal birth weight. However, approximately, more than half of infants did not meet the EAR for iron, vitamin A, and vitamin D intakes.

**Table 3:** Feeding practices at baseline and throughout the study period (*n* = 200)

Variables	Experimental Group (***n*** = 100) n(%)	Control group (***n*** = 100) n(%)	t / χ2 value	***P*** value ^**a**^
**Type of milk**				
**Exclusive breast feeding (month old)**				
0 to 5.99	100 (100)	100 (100)	−0.63	0.53
Mean ± SD	4.51 ± 0.56	4.65 ± 0.59		
**Still breastfeeding (month old)**				
At 5.99	90 (90)	96 (96)	2.76	0.09
At 11.99	68 (68)	64 (64)	0.35	0.65
At 17.99	4 (4)	3 (3)		1 †
Mean ± SD	11.57 ± 3.35	11.76 ± 2.19	−0.43	0.66
**Infant formula (month old)**				
Birth – 5.99	67 (67)	70 (70)	0.20	0.64
6–11.99	71 (71)	76 (76)	0.64	0.42
12–17.99	88 (88)	81 (81)	1.87	0.17
**Fresh milk (month old)**				
Birth – 5.99	60 (60)	56 (56)	0.32	0.56
6–11.99	47 (47)	50 (50)	0.18	0.67
12–17.99	87 (87)	93 (93)	2	0.15
**Milk powder (other than infant formula) (month old)**				
Birth – 5.99	7 (7)	5 (5)	0.35	0.55
6–11.99	67 (67)	55 (55)	3.02	0.08
12–17.99	83 (83)	90 (90)	2.09	0.14
**Others (month old)**				
Birth – 5.99	1 (1)	0 (0)		1 †
6–11.99	0 (0)	1 (1)		1 †
12–17.99	11 (11)	14 (14)	0.41	0.52
**Age at introduction of complementary foods**				
**Foods**				
**Commercial cereals**	65 (65) 6.25 ± 0.79	68 (68) 6.05 ± 0.84	1.30	0.19
**Porridge/Rice**	52 (52) 6.61 ± 0.96	55 (55) 6.77 ± 1.01	−0.76	0.44
**Biscuits/Breads**	100 (100) 5.59 ± 0.81	100 (100) 5.79 ± 0.84	−1.55	0.12
**Hen egg**	63 (63) 8.90 ± 0.6	69 (69) 9.02 ± 0.70	−0.91	0.36
**Meat/Fish**	30 (30) 12.28 ± 1.43	35 (35) 12.08 ± 1.33	0.53	0.59
**Vegetables**	90 (90) 6.89 ± 1.27	89 (89) 7.05 ± 1.34	−0.71	0.47
**Fruits**	100 (100) 7.48 ± 1.43	100 (100) 7.35 ± 1.42	0.56	0.57
**Drinks**				
**Water with sugar**	52 (52) 4.10 ± 0.62	58 (58) 4.14 ± 0.61	−0.33	0.74
**Fruit juice**	75 (75) 5.44 ± 0.85	77 (77) 5.18 ± 0.74	1.84	0.06
**Tea**	35 (35) 5.63 ± 0.84	41 (41) 5.50 ± 0.74	0.69	0.48
**Coffee**	2 (2) 12.5 ± 1.41	1 (1) 12 ± 0.00	0.28	0.82
**Others**	99 (99) 8.80 ± 0.94	96 (96) 8.87 ± 0.95	−0.46	0.64

^a^Statistical testing using independent samples t-test or chi-square test

^†^Fisher’s exact test

*Difference is significant at *p* < 0.05 (2-tailed)

**Difference is significant at *p* < 0.01 (2-tailed)

**Table 4:** Baseline characteristics of the participated infants in the two groups^b^

Characteristics	Experimental group (***n*** = 100)	Control group (***n*** = 100)	***P*** value^**a**^
**Household characteristics**			
Family size (n)	5.85 ± 2.35	5 ± 1.77	0.33
Family income (NIS)^c^	1275 ± 818.06	1000 ± 724.31	0.07
Number of under-five children (n)	2.01 ± 0.76	2 ± 0.52	0.18
**Maternal characteristics**			
Age (years)	28..41 ± 5.86	28.4 ± 5.10	0.70
Body mass index	26.54 ± 5.33	26.64 ± 4.36	0.19
Mother’s education (years)	13.18 ± 2.89	12 ± 3.01	0.54
Mother’s working status (%)			0.07
Working	6 (6)	15 (15)	
Housewives	94 (94)	85 (85)	
**Infants’ characteristics**			
Age (months)	6.28 ± 0.10	6.24 ± 0.09	0.08
Gender (%)			0.86
Male	52 (52)	51 (51)	
Female	48 (48)	49 (49)	
Birth weight (kg)	3.44 ± 0.33	3.41 ± 0.32	0.55
Gestational age (weeks)	38.31 ± 0.71	38.37 ± 0.68	0.59
Infant currently breastfeeding (%)	92 (92)	96 (96)	0.31
**Anthropometric measures**			
Weight (kg)	7.57 ± 0.79	7.65 ± 0.72	0.47
Length (cm)	66.20 ± 1.87	66.11 ± 1.92	0.75
Weight-for-age (z score)	−0.12 ± 0.92	0.01 ± 0.93	0.47
Length-for-age (z score)	−0.25 ± 0.85	− 0.28 ± 0.97	0.79
Weight-for-length (z score)	0.11 ± 1.01	0.28 ± 1.25	0.35
**Biochemical measure**			
Blood hemoglobin (g/dl)	11.42 ± 0.35	11.44 ± 0.37	0.78
**Energy and micronutrient intakes**			
Total energy (kcal/day)	656 ± 164	696 ± 167	0.11
Iron (mg/day)	7.51 ± 7.40	7.90 ± 8.55	0.75
Vitamin A (mcg/day)	299.70 ± 120.31	264.32 ± 161.19	0.10
Vitamin D (mcg/day)	3.24 ± 2.6	3.85 ± 3.03	0.16

*Difference is significant at *p* < 0.05 (2-tailed)

^a^Statistical testing using independent samples t-test or chi-square test

^b^Values are reported as means±SD

^c^*NIS* New Israeli Shekel

### Hemoglobin concentration

The hemoglobin concentrations in the two groups through the 15-month study period (12 months of supplementation and 3 months after the end of supplementation) are presented in Table 5. Over the study period, hemoglobin concentration reduced significantly in the two groups as compared to baseline (*p* < 0.05) and the reduction was significantly greater in the control group than that in the experimental group (0.29 vs. 0.52 at month 12 and 0.18 vs. 0.63 at month 15). Further, in the experimental group, hemoglobin concentration increased significantly in month 15 when compared to month 12 (*p* = 0.001), while it continued to decrease non-significantly in the control group for the same time period. A significant difference (*p* < 0.05) between the two groups was only observed in the mean hemoglobin at the end of study (month 15) with a significant group by month of assessment interaction (*p* < 0.001). After adjustment for sex, blood hemoglobin at 12 months differed significantly between the two groups, and the experimental group has significantly greater values than did the control group.

**Table 5:** Changes in anthropometric and biochemical measures by intervention group^1^

Variables	Experimental group (***n*** = 100)			Control group (***n*** = 100)			Unadjusted p Value^**a**^	Adjusted ***p*** value^**2**^
	Baseline	12 months	15 months	Baseline	12 months	15 months		
**Blood hemoglobin**								
Blood hemoglobin (g/dl)	11.42 ± 0.35	11.13 ± 0.52**	11.24 ± 0.44* ^‡ c^	11.44 ± 0.37	10.92 ± 0.61**	10.81 ± 0.59** ^c^	0.000**	0.02*
**Anthropometric measures**								
Weight (kg)	7.57 ± 0.79	11.31 ± 0.95** ^b^	12.04 ± 0.95** ^c^	7.65 ± 0.72	10.69 ± 0.82** ^b^	11.05 ± 0.80** ^c^	0.000**	0.004*
Length (cm)	66.20 ± 1.87	81.50 ± 2.56** ^b^	84.16 ± 1.99** ^c^	66.11 ± 1.92	79.93 ± 1.94** ^b^	82.12 ± 1.69** ^c^	0.000**	0.01*
WAZ (z score)	−0.12 ± 0.92	0.51 ± 0.72** ^b^	0.57 ± 0.65** ^c^	0.01 ± 0.93	0.04 ± 0.75 ^b^	−0.15 ± .0.70 ^c^	0.000**	0.003*
LAZ (z score)	−0.25 ± 0.85	−0.01 ± 0.92 ^b^	− 0.21 ± 1.33 ^c^	−0.28 ± 0.97	− 0.57 ± 0.81* ^b^	−0.78 ± 0.66** ^c^	0.001*	0.04*
WLZ (z score)	0.11 ± 1.01	0.71 ± 0.80** ^b^	1 ± 1.79* ^c^	0.28 ± 1.25	0.42 ± 0.93 ^b^	0.31 ± 0.88 ^c^	0.001*	0.02*

^1^ values are mean ± SD

^a^ Undjusted *p* values are group x time of assessment interaction and were obtained by repeated measures ANOVA analysis. Experimental group, MNP and NMS; Control group, NMS only. *WAZ* Weight-for-age z score, *LAZ* Length-for-age z score, and *WLZ* Weight-for-length z score

Difference between groups at same time point is significant (at the 0.05 level) when letters are same

^*^ Significantly different by paired t-test between baseline and selected time-point in the same intervention group at the 0.05 level

^**^ Significantly different by paired t-test between baseline and selected time-point in the same intervention group at the 0.001 level

^‡^ Significantly different by paired t test between month 12 and 15 in the same intervention group at the 0.05 level

^2^ *p* value after adjusting for sex (Model 1) at 12 months, groups were compared using one-way ANCOVA

Although the recruited infants were non-anemic at baseline, the prevalence of anemia was significantly different between the two groups at 12 months (*p* < 0.05) and 15 months (*p* < 0.001). About 29 and 18% of children in the experimental group and 54 and 52% in the control group had mild to moderate anemia at 12 and 15 months of the study, respectively (Table 6). Moreover, a significant (*p* = 0.001) and large effect size (0.84) was found for the provision of MNP on hemoglobin concentration based on the SMD.

**Table 6:** The prevalence of anemia, underweight, stunting, and wasting during the study period

Clinical indicators	Experimental (***n*** = 100) and control groups (***n*** = 100)					
	Month 12			Month 15		
	Exp. group n (%)	Control group n (%)	Total n (%)	Exp. group n (%)	Control group n (%)	Total n (%)
**Blood hemoglobin level (g/dl)**						
Normal blood hemoglobin^a^	71 (71)	46 (46)	117 (58.5)	82 (82)	48 (48)	130 (65)
Mild anemia^b^	28 (28)	48 (48)	76 (38)	18 (18)	43 (43)	61 (30.5)
Moderate anemia^c^	1 (1)	6 (6)	7 (3.5)	0 (0)	9 (9)	9 (4.5)
Severe anemia^d^	0 (0)	0 (0)	0 (0)	0 (0)	0 (0)	0 (0)
**Fisher’s exact test value**			10.74			24.20
***p*** **value**			0.003*			< 0.001**
**Weight for age (z score)**						
Normal weight for age^e^	100 (100)	95 (95)	195 (97.5)	100 (100)	97 (97)	197 (98.5)
Moderate underweight^f^	0 (0)	5 (5)	5 (2.5)	0 (0)	3 (3)	3 (1.5)
Severe underweight^g^	0 (0)	0 (0)	0 (0)	0 (0)	0 (0)	0 (0)
**Fisher’s exact test value**			3.81			2.84
***p*** **value**			0.12			0.24
**Length for age (z score)**						
Normal length for age^e^	100 (100)	91 (91)	191 (95.5)	99 (99)	89 (89)	188 (94)
Moderate stunting^f^	0 (0)	9 (9)	9 (4.5)	1 (1)	11 (11)	12 (6)
Severe stunting^g^	0 (0)	0 (0)	0 (0)	0 (0)	0 (0)	0 (0)
**Fisher’s exact test value**			7.82			11.89
***p*** **value**			0.007*			0.002*
**Weight for length (z score)**						
Normal weight for length^e^	100 (100)	98 (98)	198 (99)	100 (100)	98 (98)	198 (99)
Moderate wasting^f^	0 (0)	2 (2)	2 (1)	0 (0)	2 (2)	2 (1)
Severe wasting^g^	0 (0)	0 (0)	0 (0)	0 (0)	0 (0)	0 (0)
**Fisher’s exact test value**			1.88			1.88
***p*** **value**			0.49			0.49

*NB* At baseline, the prevalence of anemia, underweight, stunting, and wasting was zero

* *p* < 0.05; ** *p* < 0.001 using Fisher’s exact test

^a^ Hemoglobin: ≥ 11 Gram/deciliter, ^b^: 10 to 10.9 Gram/deciliter, ^c^: 7 to 9.9 Gram/deciliter, ^d^: < 7 Gram/deciliter

^e^ Normal underweight, stunting, or wasting: ≥ −2 SD of the WHO median for WAZ, LAZ, or WLZ

^f^ Moderate underweight, stunting, or wasting: ≥ −3 SD but < −2 SD of the WHO median for WAZ, LAZ, or WLZ

^g^ Severe underweight, stunting, or wasting: < −3 SD of the WHO median for WAZ, LAZ, or WLZ

### Anthropometric measures

The mean anthropometric values obtained during the study period (12 months of supplementation and 3 months after the end of supplementation) are presented in Table 5. All anthropometric indices increased significantly (*p* < 0.05) in the two groups from baseline to month 12 and 15, except for LAZ in the experimental and control groups, in addition to WAZ and weight-for-length z score (WLZ) in the control group. As compared to baseline, LAZ in the experimental group and WAZ, and WLZ in the control group were non-significantly increased and decreased at month 12 and 15, respectively (*p* > 0.05). Further, LAZ worsened significantly from baseline to month 12 and 15 in the control group (*p* < 0.05). The increment in weight, length, WAZ, and WLZ in the experimental group was significantly higher than that in the control group at 12 and 15 months as compared to baseline. Although LAZ increment in the experimental group was greater than that in the control group at 12 and 15 months, it did not reach a level of significance. Significant differences were observed between the two groups in the mean anthropometric measures (*p* < 0.05) with a significant group by month of assessment interaction (*p* < 0.01). After adjustment for sex, anthropometric measures at 12 months differed significantly between the two groups, and the experimental group has significantly greater values than did the control group (Table 5).

Although all infants had normal weight-for-age, and length-for-age z scores at the beginning of the study, the prevalence of underweight and wasting in the control group was insignificantly higher than that in the experimental group at month 12 and 15 [underweight; (5% vs. 0%) and (3% vs. 0%), wasting; (2% vs. 0%) and (2% vs. 0%), respectively]. Furthermore, a significant higher prevalence of stunting (*p* < 0.05) was observed in the experimental group than that in control groups at 12 months (0% vs. 9%) and 15 months (1% vs. 11%), respectively (Table 6). Moreover, the findings of the current study showed a significant (*p* < 0.05) and medium to large effect size for provision of MNP on participants’ weight-for-age (SMD = 1.07), length-for-age (SMD = 0.95), and weight-for-length (SMD = 0.49).

### Energy and micronutrient intakes

Table 7 showed the changes in mean energy and micronutrients (NMS and MNP doses were excluded) intakes during the supplementation period. As compared to baseline values, mean values for energy, vitamin A, and vitamin D intakes increased significantly in the two groups (*p* < 0.05). However, iron intake decreased significantly in the two groups as compared to the baseline intake (*p* < 0.05). Significant differences between the two groups were only observed in the mean vitamin A and vitamin D intakes after 6 months of supplementation with a significant group by month of assessment interaction for vitamin D intake (*p* < 0.05). Similar findings were also reported after adjustment for sex at 12 months.

**Table 7:** Changes in energy, iron, vitamin A, and vitamin D intakes by intervention group ^1^

Dietary factors	Experimental group (***n*** = 100)			Control group (***n*** = 100)			Unadjusted ***p*** value ^**a**^	Adjusted ***p*** value ^**2**^
	Baseline	6 months	12 months	Baseline	6 months	12 months		
Energy (kcal)	655 ± 164	784 ± 163**	818 ± 111**	696 ± 167	751 ± 114	835 ± 138**	0.06	0.21
Iron (mg)	7.51 ± 7.40	5.96 ± 7.31	3.35 ± 1.94**	7.90 ± 8.55	5.84 ± 5.18	3.28 ± 1.66**	0.88	0.61
Vitamin A (RAE, mcg)	299.7 ± 120.3	409.7 ± 297.2* ^b^	362.37 ± 119.08*	264.32 ± 161.1	340.04 ± 114.80* ^b^	367.71 ± 224.24*	0.15	0.32
Vitamin D (mcg)	3.24 ± 2.60	5.19 ± 3.62* ^c^	6.97 ± 3.44**	3.85 ± 3.03	6.98 ± 3.27** ^c^	6.45 ± 2.67**	0.003*	0.02*

^1^ values are mean ± SD

^a^ *p* values are group x time of assessment interaction and were obtained by repeated measures ANOVA analysis

Experimental group, MNP and NMS; Control group, NMS only

Difference between groups at same time point is significant (at the 0.05 level) when letters are same

^*^ Significantly different by paired t-test between baseline and selected time-point in the same intervention group at the 0.05 level

^**^ Significantly different by paired t-test between baseline and selected time-point in the same intervention group at the 0.001 level

^2^ *p* value after adjusting for sex (Model 1) at 12 months, groups were compared using one-way ANCOVA

## Discussion

The first 2 years of life is a crucially important period, and adequate nutrition through this time is fundamental for growth and development of child to its fullest potential [34]. Studies have demonstrated adverse consequences of inappropriate dietary intake and practices on nutritional status of infants and children [35, 36]. The present study examined the effect of MNP (3 sachets/week) combined with NMS administered for a 12-month period on the nutritional status of Palestinian infants.

Although hemoglobin concentration worsened significantly in the two groups as compared to baseline, the present study showed that hemoglobin concentration was significantly higher in the experimental group than that in the control group at 3 months after the end of supplementation, and a significant increase in mean hemoglobin at the end of the study was reported in the experimental group when compared with 12 months of the supplementation. Moreover, the experimental group had a significantly lower prevalence of anemia throughout the study as compared to the control group.

In a comprehensive review of nutrition situation of Gaza Strip, mothers reported differences in frequency and amount of micronutrient drops given, highlighting poor compliance [37]. Therefore, although the absorption of iron from the ferrous sulphate used in the NMS is 2–3 times greater than the ferrous fumarate used in the MNP [38], low compliance to the NMS in Gaza Strip could explain the worst hemoglobin change in the control group. In contrast, the non-significant change in hemoglobin throughout the intervention period in the experimental group could be explained by the inclusion of zinc with iron in the MNP which could have reduced absorption due to zinc and iron interaction and may affect the results [39]. Consistent with a 12-month cluster randomized community trial in Pakistan [18]; the 6-months aged non-anemic children who had iron intake less than recommended were supplemented with MNP with 10 mg zinc, MNP without zinc or placebo daily for 12 months showed a significant decrease in the mean hemoglobin concentration between the experimental and control groups, and the decrease in hemoglobin concentration in the control group (0.7 g/dl) was more than that in the experimental groups (0.3 to 0.4 g/dl). It should be pointed out that 55% of infants in this study had iron intake less than the EAR at baseline, and a significant correlation was reported between iron intake of children at 18 months old and hemoglobin concentration at 21 months old (*p* = 0.01). Furthermore, the iron dose used in the current study was lower than the WHO recommended dose of 12.5 mg/day for children under two [40] due to the concern that higher doses of iron supplements consumed for 12 months could produce adverse effects [41]; however, no adverse event was recorded.

Nevertheless, a significant large effect size for addition of MNP on hemoglobin concentration was reported in the present study. Similarly, a significant small to large effect size were reported in a review study (SMD = 0.98) [42] and a meta-analysis (SMD = 0.39) [43]. Factors such as age (younger vs. older children) and population (low-income vs. high-income population) groups, duration of supplementation and dosage of micronutrients could explain the different outcomes, suggesting that micronutrients supplementation would have a greater effect if a well-established dosage and length of supplementation was considered among young children.

Administration of MNP three sachets per week for 12 months with NMS resulted in an overall improvement of most anthropometric measures and lower undernutrition prevalence than use of NMS alone. This supports the results of other micronutrients supplementation trials which suggest that MNP supplement was effective for improving anthropometric indices and underweight, stunting, and wasting prevalence [13, 18]. The limited age range of children in the present study (6 to 18 months), and high compliance to supplementation regimen which was satisfactory (91%) could contribute to the observed improvement. Rivera et al. indicated that the supplementation effect with high compliance rate 86% on length gain during the first years of life demonstrated the highest benefit [44]. The greater effect on young children (less than 2 years) than older children is biologically plausible. Under-two children grow at a faster rate than older children, their micronutrient needs to sustain this accelerated growth are greater, and their diets are often more restricted than those of older children. Therefore, most of the supplementary feeding effect occurs during the first 2 years of life [45]. However, the findings of meta-analysis to assess the effect of fortification of home food with micronutrient powders on the nutritional status of healthy children aged 6 to 23 months did not report an impact on growth. The possible rationale for difference between results could be due to different formulations of the micronutrient powders, different duration of supplementation (ranged from 2 to 12 months), different compliance rate, and some of the children were apparently healthy but at risk of having a highly prevalence of diseases including malaria, diarrhea or other undernutrition forms [21].

In term of effect size, a significant medium to large effect size for the provision of MNP on weight-for-age, length-for-age, and weight-for-length was reported in the current study. In the line of this study, a meta-analysis including 16 micronutrients supplementation studies (5 anemia treatment trials, and 11 prevention trials and duration from 2 to 13 months) showed a significant small to medium effect size for MNP with small amount of energy on weight-for-age (SMD = 0.39), length/height-for-age (SMD = 0.41), and weight-for-length (SMD = 0.12). However, a non-significant small effect size for MNP without energy on weight-for-age (SMD = 0.08), length/height-for-age (SMD = 0.02), and weight-for-length (SMD = 0.01) was reported in the same review [46]. Few studies are available for the effect size comparison [46], but these findings supported the contention that home fortification with supplements that include some energy would be more effective particularly among unhealthy or highly risk children and programs with short duration regimens.

### Limitations of the study

The present study had several limitations, including inability to use placebo or keeping participants blind to the type of supplement due to logistical, and social constrains, respectively. However, outcomes being measured were objective and duplicate assessment of outcomes was considered. Although attrition could have introduced selection bias, there was no significant difference between children who remained in the study and those who dropped out which dismissed that possibility. Finally, the present study used a rigorous design (randomized controlled trial) with many potential confounders were assessed as well as intention to treat analysis.

## Conclusion

The results of the present study suggest that starting MNP supplements at the age of 6 months, when human milk alone fails to meet the dietary requirements and complementary foods of poor nutritional value are introduced, may be promising in improving the nutritional status during the complementary feeding period and thereafter. Particularly, in infants at risk of undernutrition living in developing countries such as Palestine, where common complementary foods are poor in nutritive value and the NMS program distributed by the Ministry of Health is inadequate. In addition, micronutrient powder demonstrated a prophylactic effect in the present study which suggested that modifying the dose or/and the duration of MNP intake may have possible effects on initially undernourished children, but further studies are required.

## Data Availability

Not Applicable.
